# Comparative analysis of the microbial community and nutritional quality of sufu

**DOI:** 10.1002/fsn3.2372

**Published:** 2021-06-23

**Authors:** Xingjiang Li, Ying He, Wei Yang, Dongdong Mu, Min Zhang, Yilong Dai, Zhi Zheng, Shaotong Jiang, Xuefeng Wu

**Affiliations:** ^1^ Key Laboratory for Agricultural Products Processing of Anhui Province School of Food and Biological Engineering Hefei University of Technology Hefei, Anhui Province China; ^2^ Tianjin Agricultural University Tianjin China; ^3^ Anhui Bagongshan Bean Foods Product Co. Shouxian China

**Keywords:** amino acids, fatty acids, microbial, nutritional, Sufu

## Abstract

Sufu is a type of fermented food with abundant nutrients and delicious taste. It is made from the fermentation of tofu by various microorganisms. In this study, three types of sufu were prepared through natural fermentation: (NF), single‐strain fermentation (SF), and mixed‐strain fermentation (MF). Microbial species, amino acids, and fatty acids were identified to investigate dynamic changes in nutritional quality and microbial flora in sufu. The results showed that the number of microbial species in NF sufu was the highest (*n* = 284), whereas that in SF sufu was the lowest (*n* = 194). Overall, 153 microbial species were found in all three types of sufu. Relative abundance analysis also revealed that *Tetragonococcus*, *Bacillus*, *Acinetobacter*, and *Staphylococcus* were the main bacteria in sufu. However, there was a large number of harmful bacteria such as *Enterococcaceae* in NF sufu. The levels of various nutrients were low in SF sufu, whereas the contents of protein and soy isoflavones were higher in NF and MF sufu. Seventeen kinds of amino acids were detected, comprising seven essential amino acids and ten other amino acids. The contents of essential amino acids and essential fatty acids were higher in MF sufu than the other two types, resulting in its high nutritional value. The sufu produced through the three fermentation methods differed significantly (*p* < .05) in terms of microbial flora and nutritional quality.

## Graphical abstract

The microbial communities and nutrient contents in sufu were analyzed. There are differences in microbial community and nutrients in sufu. Sufu from a mixed fermentation had the best nutritional qualities.

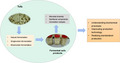

## 1 INTRODUCTION

Sufu is a traditional Chinese fermented food that is made from the fermentation of tofu by various microorganisms (Canonico et al., 2018; Guan et al., 2013). Traditionally, to produce sufu, tofu is fermented by various types of mold or bacteria from the natural environment. Currently, with the development of biotechnology, some specific molds are selected for the large‐scale production of sufu, which shortens the fermentation time. Although many types of microorganisms are used for the fermentation of sufu, *Mucor* fungi are currently mainly selected to produce sufu in China. Many types of enzymes with high activity are involved in the fermentation of tofu by *Mucor* fungi, and these enzymes play important roles in promoting the maturation of sufu (Tang et al., 2011). The disadvantage of using *Mucor* in sufu production is their low growth temperature. The growth of *Mucor* is inhibited above 28℃. Sufu cannot be produced when the environmental temperature exceeds 28℃. Recently, the *Rhizopus oryzae* fungus has been used in the production of sufu; because of its high‐temperature tolerance, it can grow favorably at temperatures close to 40℃ (Ma et al., 2015).

Sufu is rich in numerous nutrients in addition to those found in tofu. After fermentation, the protein and starch in tofu are decomposed into amino acids, peptides, and other nutrients by fungal enzymes, which can give a unique flavor to sufu. At the same time, the bitter taste, gas‐producing effect, and anti‐nutritional properties of soybeans were weakened, and the production of soy isoflavones was enhanced. The concentrations of antioxidants and active substances as well as biovalence are also greatly increased (Xu et al., 2015). In the fermentation process, bacterial strains produce peptidase or protease, which can effectively decompose the macromolecules in tofu into small‐molecule substances. These small‐molecule substances are easily absorbed by the human body (Chen et al., 2016; González et al., 2018).

The microbial flora in fermented foods determines their safety, odor, and nutritional quality. Many studies have investigated the nutrients in sufu. However, due to the complexity fermentation process of sufu, how its nutritional quality is affected by the microflora remains unclear (Lv et al., 2015). Traditionally, sufu is produced through natural fermentation (NF). Currently, *Mucor* are usually used in single‐strain fermentation (SF). However, NF sufu contains a multitude of microorganisms, even including many pathogenic bacteria. In the SF production process, the overgrowth of a single strain inhibits the growth and reproduction of other beneficial bacteria, which affects the nutritional quality of sufu. In order to enrich the nutrients and improve the odor of sufu, in this study, NF sufu, SF sufu, and mixed‐strain fermentation (MF) sufu were produced using different microorganisms, and the bacterial population and nutrient changes were analyzed.

## 2 MATERIALS AND METHODS

### 2.1 Preparation of fungi

*Mucor racemosus* (CICC40481) and *R. oryzae* preserved in potato dextrose medium were washed with 20 ml of sterile water and filtered thorough sterile gauze. The filtrate was collected, and cells were counted using a hemocytometer. The spore suspension concentration was adjusted to 10^6^ (cfu/mL) as the seed.

### 2.2 Sample preparation

Soybeans were screened, soaked, milled, filtered, boiled, spotted, and chopped into 2.8 × 2.8 × 1.4‐cm^3^ white bars. The water content of the white bars was controlled at approximately 70% (Qiu et al., 2018), and each white bar was gently placed in a salver. The white bars were fermented under natural conditions without any seed in NF, while *Mucor* spore suspension was dropped on the white bars in SF and a mixed suspension of *Mucor* and *R. oryzae* spore with a ratio (1.5﹕1) was dropped on the white bars in MF (Feng, Gao, Ren, Chen, & Li, 2013). These samples were maintained in an environment with 28℃ temperature and 90% relative humidity for two days. After the surface of the white bars was covered with mycelium, they were marinated with salt and then packaged into 300‐mL glass bottles, which were filled with a solution of 12% ethanol and each auxiliary material. Finally, the bottles were aged at room temperature for 90 days to obtain sufu (Han et al., 2004) (Figure B1 and Figure B2).

### 2.3 DNA extraction, amplicon, and sequencing

In this study, 5.0 g of sufu was mixed with 25 ml of phosphate buffer (pH 7.2). The mixture was centrifuged at 5,000 rpm for 10 min, and then, 5 ml of the supernatant was subjected to genomic DNA extraction using a DNA kit (Sangon Biotech, Shanghai) (Almeida et al., 2018; Knob et al., 2018). The extracted genomic DNA was separated through 1% agarose gel electrophoresis. The V3–V4 domain of the 16S rRNA gene was amplified using the primers 338F and 806R (Table A1, ABI, Foster City, CA, USA) using TransGen AP221‐02 (TransStart Fastpfu DNA Polymerase) and a PCR machine (ABI GeneAmp Model 9700). Three replicates of each sample were prepared. The PCR products of each sample were mixed and detected using 2% agarose gel electrophoresis (Jarocki et al., 2016). The PCR products were recovered using the AxyPrep DNA gel recovery reagent and were eluted with Tris‐HCl before undergoing 2% agarose electrophoresis. With reference to the preliminary electrophoretic quantification results, the PCR products were quantified using a QuantiFluorTM‐ST blue fluorescence quantitation system (Promega) (Naegele et al., 2018). Amplicons were submitted to Majorbio Bio‐Pharm Technology Co., Ltd. (Shanghai, China) for Illumina paired‐end library preparation, cluster generation, and 300–500 bp paired‐end sequencing on the MiSeq instrument.

### 2.4 Data management and species annotation and assessment

MiSeq sequencing results were obtained from double‐ended sequence data. According to the overlap relationship between PE reads, the paired reads were merged into a sequence, and the quality of the reads and the effects of the merged sequence were quality‐control‐filtered on the basis of the barcodes at each end of the sequence. Primer sequences were used to distinguish the samples to obtain valid sequences and the correct orientation of the sequence for generating optimized data. Clustering of the sequences was conducted at 97% similarity levels, and one group contained OTU representative sequences (Gryganskyi et al., 2018). Taxonomic analysis of the samples was conducted at 97% similarity levels using the RDP classifier Bayesian algorithm and OTU representative sequences. The samples were compared with the sequences in the SILVA database, and the statistics of each sample at each taxonomic level were used to determine the community composition. One‐way analysis of variance was used to detect significant differences. Fisher's exact test was used to determine the differences in the abundance of two fermented sufu species across two or three sufu samples (Kulkarni et al., 2018).

### 2.5 Nutritional quality analysis

The sufu sample was weighed (5.0 g, accurate to 0.001 g) in a filter paper cylinder. The filter paper cylinder was placed in a Soxhlet extraction tube for Soxhlet extraction. After the extraction, the fat content was calculated using the dry weight. Phenolphthalein was then added to a receiving flask, and then titration was performed with a 10% sodium hydroxide solution. The volume of sodium hydroxide solution consumed was recorded to calculate the fatty acid content (Rusu et al., 2018).

The sufu sample (2.0 g, accurate to 0.001 g) was accurately weighed in Kjeldahl bottles, and 0.5 g of a copper sulfate and potassium sulfate mixture was added, followed by 12 ml of concentrated sulfuric acid. The sample was placed on a digestion rack at a temperature of 400℃. After 3–4 hr of digestion, the crude protein content in the sample was determined on an automated Kjeldahl nitrogen analyzer.

A high‐performance U3000 liquid chromatograph (Thermo Fisher) was used to determine the amino acid content. Total amino acids (TAAs) were determined under the following chromatographic conditions: mobile phase A was 0.1 mol/L sodium acetate solution and mobile phase B was acetonitrile‐water (8:2), a 10 cm column with octadecylsilane‐bonded silica gel as a filler (4.6 × 250 mm, 5 μm) was used, the flow rate was 1.0 ml per minute, the column temperature was 40℃, the injection volume was 10 μL, and the wavelength was 254 nm (Liu, Han, Deng, Sun, & Chen, 2018). The lyophilized samples were then homogenized and dissolved in sulfosalicylic acid, and free amino acid (FAA) detection was performed using sodium citrate buffer systems and ninhydrin detection columns (pH 2.2, 3.3, 4.3, and 5.4) (Mocan et al., 2018).

## 3 RESULTS

### 3.1 Analysis of microflora in sufu

**FIGURE 1:**
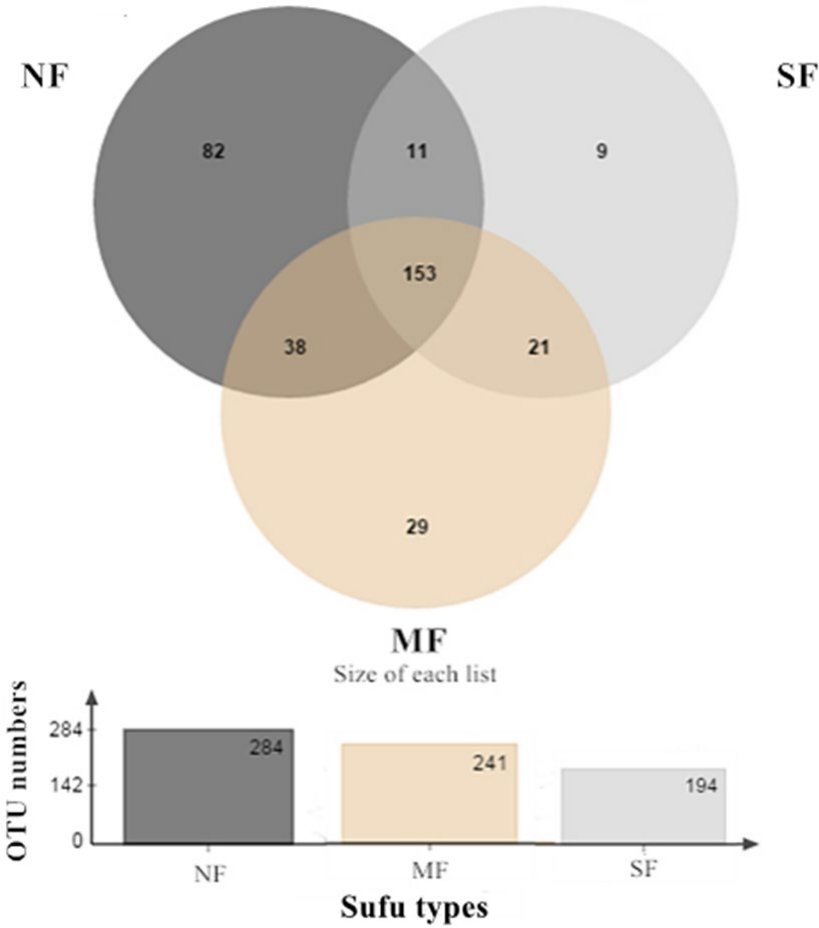
Venn diagram analysis of the numbers of shared and unique species of bacteria in sufu. NF: Natural fermentation, MF: Mixed fermentation, SF: Single strain fermentation

In Figure 1, the Venn diagram presents the numbers of common and unique bacterial species (at the species and genus levels) in sufu. Different colors represent different sufu samples produced using the various fermentation methods (NF, MF, and SF). Nonoverlapping parts represent the unique species in each sufu type, and the numbers denote the corresponding number of species. As shown in Figure 1, 153 species were found in all three types of sufu, while 191 (38 + 153) species were found in both NF and MF sufu, 164 (11 + 153) species in NF and SF sufu, and 174 (21 + 153) species in MF and SF sufu. Compared with SF and MF sufu, NF sufu had the highest numbers of total and unique species (284 and 82, respectively).

**FIGURE 2:**
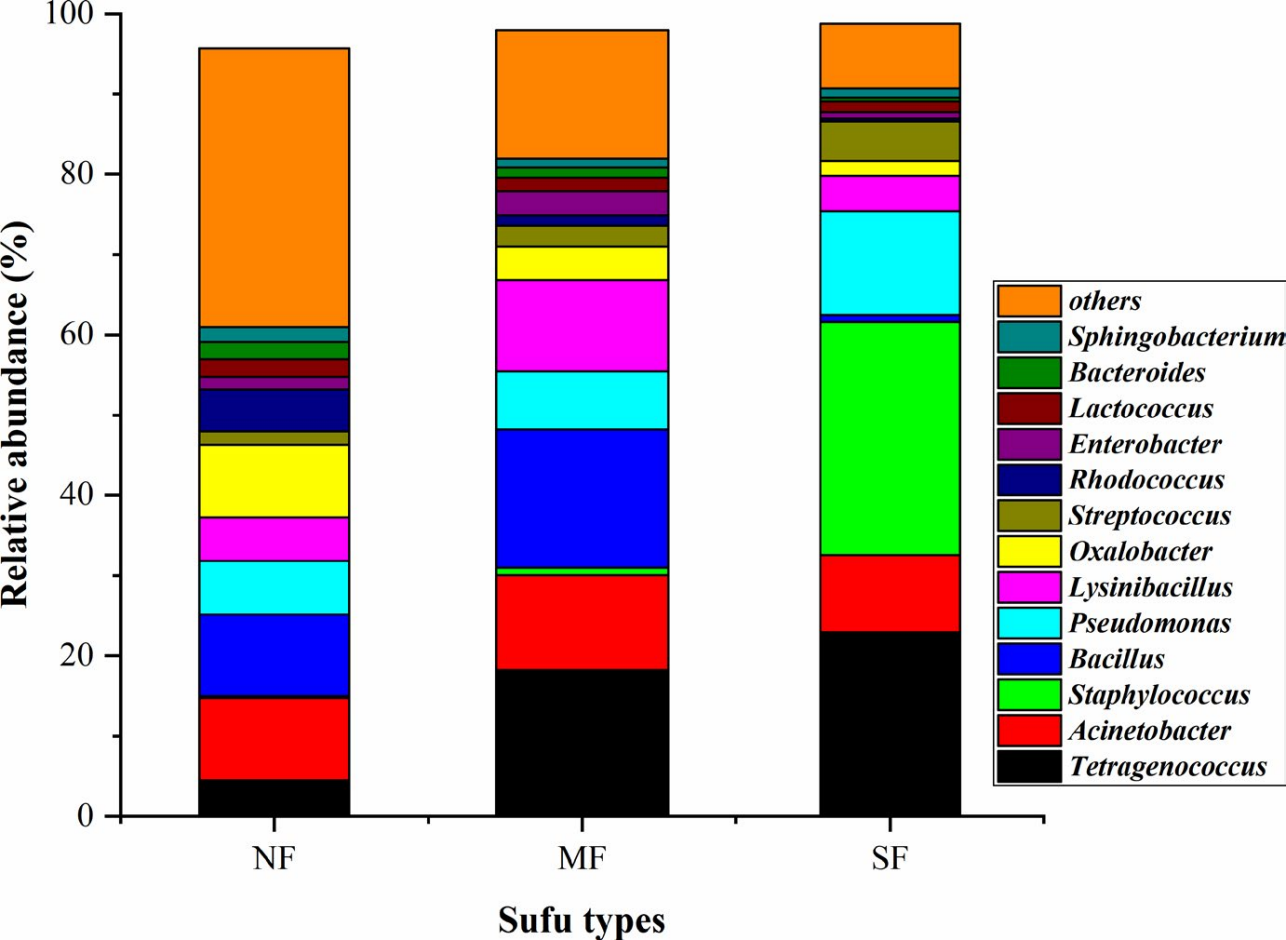
Analysis of the bacterial community composition in sufu. NF: Natural fermentation, MF: Mixed fermentation, SF: Single strain fermentation. The ordinate is relative abundance of the species in sufu, the abscissa is the sufu types; the columns of different colors represent different species, and the length of the column represents the relative abundance of the species

The sequencing reads of bacteria were classified at the genus level. The areas with different colors in the histogram represent the abundance of different species (Figure 2 and Figure A1); species with an abundance of less than 1% are also presented in the histogram. The results showed that 13 dominant bacteria were detected in each sufu, including *Bacillus*, *Tetragenococcus*, *Acinetobacter*, *Pseudomonas*, and *Staphylococcus* (relative abundance greater than 1%). The results revealed significantly different composition of bacteria communities in NF, MF, and SF sufu (*p* <.05). Among these bacteria, *Acinetobacter* and *Bacillus* were predominant in NF sufu; *Tetragenococcus* and *Bacillus* were predominant in MF sufu, and *Staphylococcus* and *Tetragenococcus* were predominant in NF sufu. Among them, *Bacillus* was predominant in all three types of sufu, especially in MF sufu (with the highest relative abundance of approximately 20%). This indicated that *Bacillus* species probably play key roles in sufu production. *Staphylococcus* was also predominant in all three types of sufu, especially in SF sufu (with the highest relative abundance of approximately 30%). Similar to *Enterococcu*s, some members of *Staphylococcus* are pathogenic. (Jung et al., 2016). Therefore, more research attention should be paid to their roles in sufu production in the future.

### 3.2 Analysis of species in sufu

**FIGURE 3:**
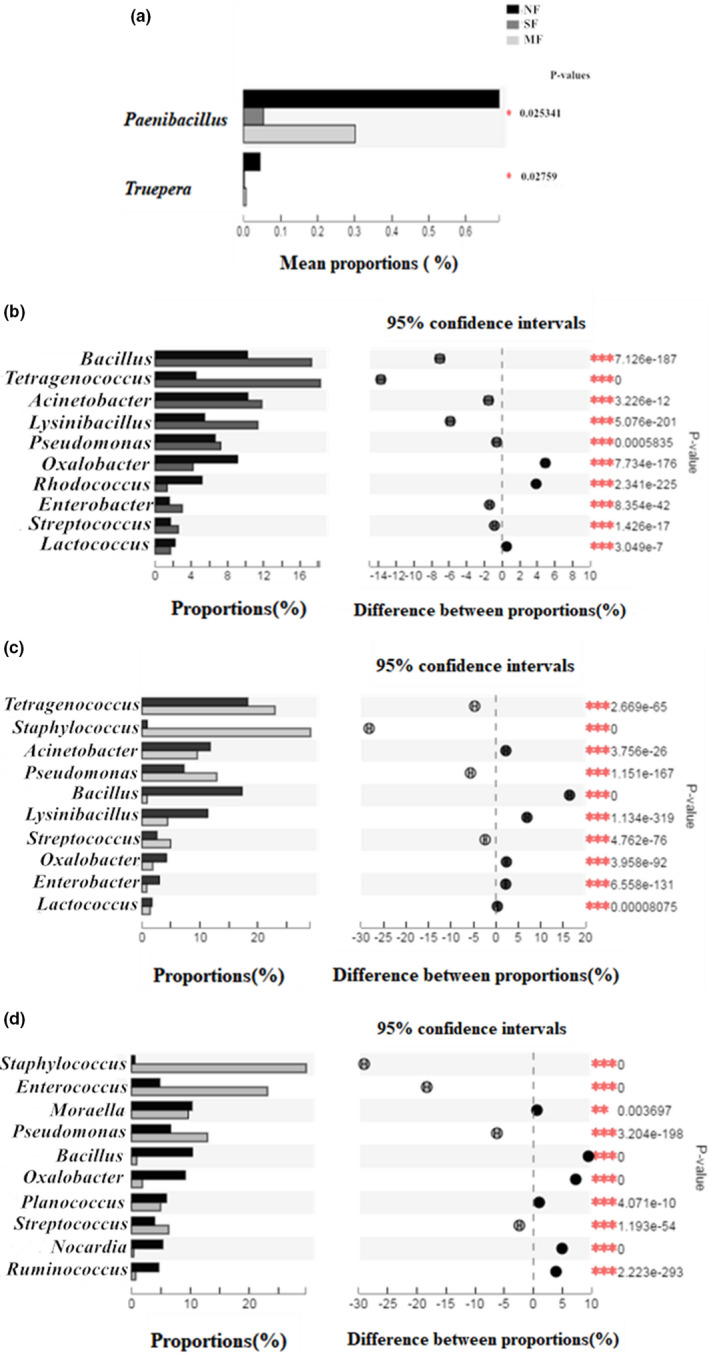
Analysis of species differences in sufu. NF: Natural fermentation, MF: Mixed fermentation, SF: Single strain fermentation. (a) Species difference analysis among NF, SF and MF sufu; (b) Species difference analysis between NF and SF sufu; (c) Species difference analysis between SF and MF sufu; (d) Species difference analysis between NF and MF sufu. One‐way analysis of variance was performed among three types of sufu. The vertical axis represents the species name under the genus level. The column length corresponds to the relative abundance of the species in each sample. Different colors indicate different samples. P‐values: * 0.01 < *p*≤.05, ** 0.001 < *p*≤.01, and *** *p* ≤.001

STAMP analysis and Welch's *t* test revealed the top 10 species in NF, MF, and SF sufu. Only *Paenibacillus* and *Truepera* were significantly different (*p* <.05) among the top 10 species (Figure 3a). The abundance of *Paenibacillus* in NF sufu was significantly higher (*p* <.05) than that in MF and SF sufu (Figure 3a). As shown in Figure 3, an analysis of species in the sufu types showed that there were significant differences in the proportions of microbial populations of NF and SF Sufu (Figure 3b), SF and MF Sufu (Figure 3c), and NF and MF Sufu (Figure 3d) at the genus level (*p* <.05). The abundance of *Lactococcus*, *Rhiodococcus*, and *Oxalicobacter* in NF sufu was significantly higher (*p* <.05) than that in SF sufu (in Figure 3b). The abundance of *Acinetobacter*, *Bacillus*, *Lysinibacillus*, *Oxalicobacter*, and *Enterococcus* in SF sufu was higher than that in MF sufu (Figure 3c). Moreover, the abundance of *Enterococcus*, *Staphylococcus*, *Pseudomonas*, and *Streptococcus* in MF sufu was higher than that in NF sufu (Figure 3d). These results accorded with the dynamic changes in the bacteria community of sufu (Figure 2). During sufu fermentation, bacteria metabolism varies depending on the environment and fermentation time.

### 3.3 Analysis of nutritional quality in sufu

**FIGURE 4:**
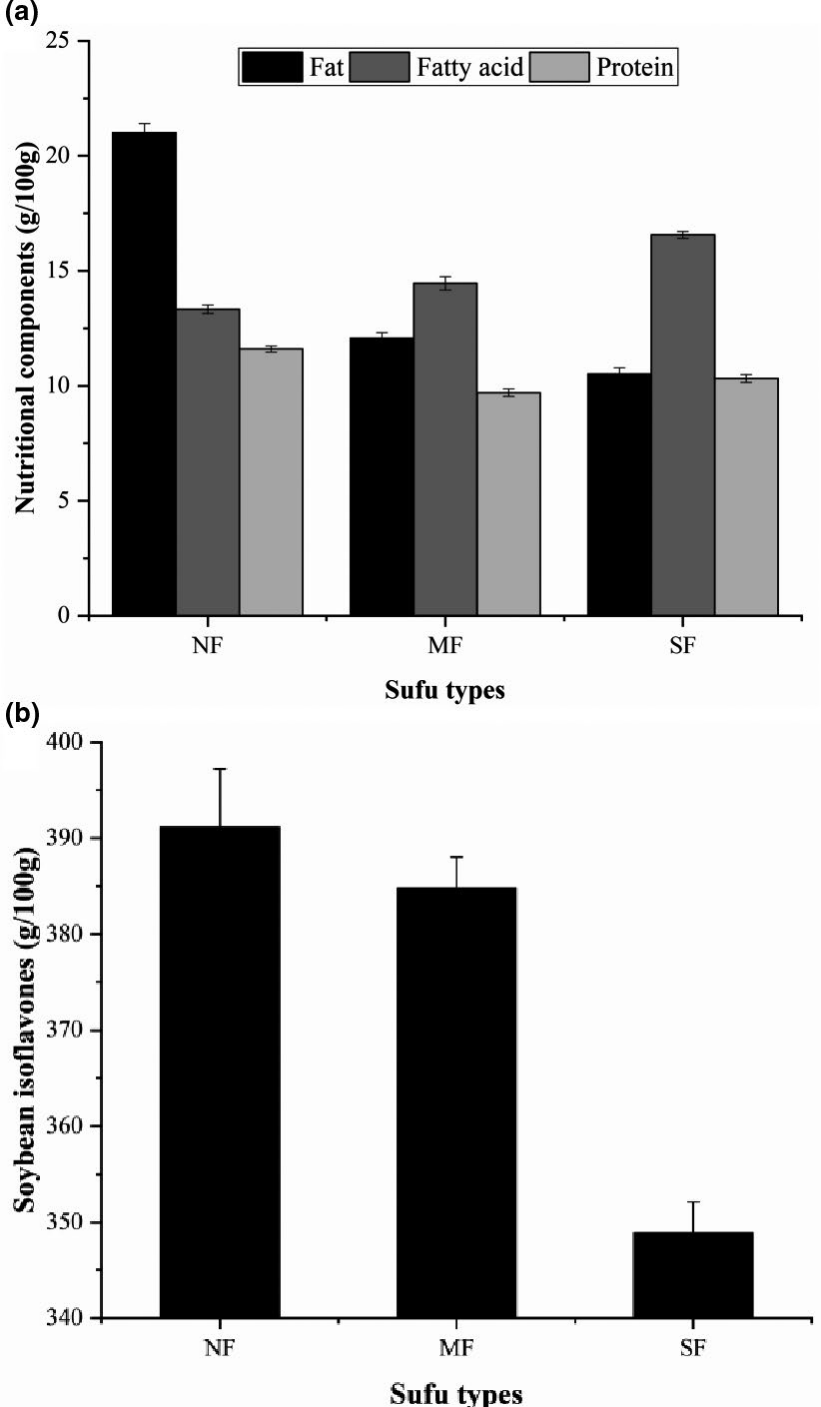
Analysis of various nutrients in sufu. NF: Natural fermentation, MF: Mixed fermentation, SF: Single strain fermentation. (a) Content of fat, fatty acid and protein in sufu; (b) Content of soybean isoflavones in sufu

As shown in Figure 4a, the fat content in NF sufu (21.38 g/100 g) was much higher than the fat contents in MF (12.02 g/100 g) and SF sufu (10.08 g/100 g). The fatty acid content in SF sufu was the highest (16.73 g/100 g). The overall protein content in the three types of sufu was the same, and the average content was approximately 10%. Fat and protein are not only important nutritional components in sufu but also important indicators of the maturity of sufu during fermentation. The soy isoflavone content was the same in NF and MF sufu; in comparison, it was slightly lower in SF sufu (Figure 4b). Texture analysis results and scanning electron microscopy images of the structure of sufu during fermentation are shown in Figure A2 and Figure A3. The results showed that approximately 30 hr after inoculation, the hardness of sufu reached the maximum level and then decreased (Figure A2). The hardness of NF sufu was slightly higher than that of SF and MF sufu. The adhesiveness of sufu increased gradually during fermentation. MF sufu exhibited the least change in elasticity. There was no significant difference among the texture analysis. But differences in structure were observed in three types of sufu. As shown in Figure A3 the structure in SF sufu was looser than that in NF and MF sufu.

**FIGURE 5:**
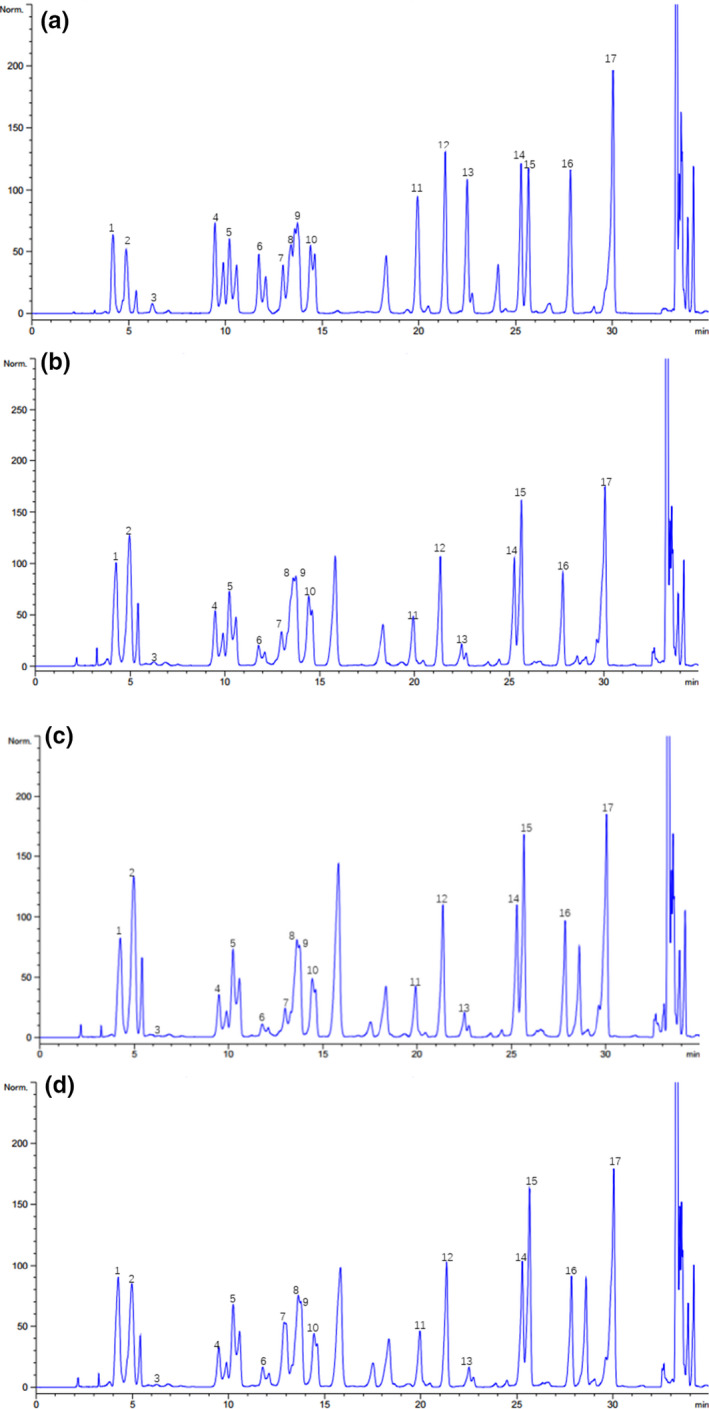
Amino acid analysis in sufu. (a) Standard amino acids, (b) Amino acid analysis of NF sufu, (c) Amino acid analysis of MF sufu, (d) Amino acid analysis of SF sufu

Amino acids are important flavor substances, especially FAAs, which are closely related to the development of the unique flavor of sufu. A total of seventeen different kinds of amino acids were detected through liquid chromatography, including seven essential amino acids (EAAs) and ten other amino acids, excluding tryptophan. The amino acid chromatograms of the three types of sufu are shown in Figure 5. Based on the standard protein map (Figure 5a), the peak area was used to calculate the TAA content in sufu. The results are shown in Table 1. The TAA content in NF sufu was the highest, at approximately 293.73 g/kg. However, the contents of EAAs such as lysine (Lys), leucine (Leu), valine (Val), isoleucine (Ile), and phenylalanine (Phe) in MF sufu were higher than those in NF and SF sufu. Among these amino acids, glutamate (Glu) had the highest concentration, reaching 61.16 g in MF sufu (per 1.0 kg sufu). Glu is responsible for sufu's umami flavor. The results of FAA analysis are presented in Table 2. In SF sufu, the FAA content was higher than that in NF and MF sufu, and the EAA content was also significantly higher (*p* <.05) in SF sufu than in NF and MF sufu.

**TABLE 1:** TAA contents of sufu

							(g/kg)
TAA	NF	MF	SF	EAA	NF	MF	SF
Asp	34.79	30.00	33.25	Lys	21.77	22.19	22.32
Glu	56.31	61.16	41.35	Leu	23.73	25.81	25.53
Cys	6.64	2.25	4.81	Thr	20.84	20.05	15.28
Ser	14.60	10.12	9.84	Val	14.38	16.32	15.42
Gly	13.05	13.70	13.19	Met	4.09	4.14	3.25
His	7.76	4.76	6.64	Ile	15.44	17.04	16.55
Arg	10.05	7.52	41.29	Phe	15.91	17.87	17.30
Ala	7.12	5.93	4.98	subtotal	116.16	123.42	115.65
Pro	15.37	11.51	10.45				
Tyr	11.88	10.87	11.20				
Subtotal	177.57	157.82	177				

**TABLE 2:** FAA contents of sufu

							(g/kg)
FAA	NF	MF	SF	EAA	NF	MF	SF
Asp	0.76	0.69	0.71	Lys	0.77	0.79	0.81
Glu	1.29	1.51	1.76	Leu	0.53	0.71	1.03
Cys	0.13	0.14	0.25	Thr	0.64	0.95	1.28
Ser	2.26	1.60	1.12	Val	1.78	1.32	1.42
Gly	1.26	1.05	1.70	Met	1.79	2.14	3.25
His	0.86	0.76	0.76	Ile	1.44	1.74	1.55
Arg	1.54	0.95	0.52	Phe	0.91	1.17	1.30
Ala	0.20	0.72	1.93	subtotal	7.86	8.82	10.64
Pro	1.54	3.37	3.51				
Tyr	0.48	0.88	0.87				
Subtotal	10.32	11.67	13.13				

## 4 DISCUSSION

In this study, the sufu produced through three fermentation methods exhibited significant differences (*p* <.05) in terms of bacterial species and nutritional quality. NF sufu contained the largest number of bacterial species (*n* = 284), whereas SF sufu had the least number of bacterial species (*n* = 194). *Bacillaceae* and *Staphylococcaceae* belong to the *Bacillales* order. *Bacillus* and *Staphylococcus* belong to the *Firmicutes* phylum. *Bacillus* can produce a variety of digestive enzymes, which can enhance the digestion and absorption of nutrients. The activities of protease, amylase, and lipase are high in *Bacillus* (Cha et al., 2018). In the current study, the *Bacteroides* phylum and the *Firmicutes* phylum were found in MF sufu. The enzyme activity of the species of the *Firmicutes* phylum is higher than that of the species of the *Bacteroidetes* phylum, which enables the more effective absorption of nutrients from food, reducing the risk of obesity. Wang et al. detected that the purine content of fermented sufu was high, which was related to *Acinetobacter* (Wang et al., 2018). Gout is a purine metabolism disorder, so patients with gout should not eat sufu. *Enterococcaceae* is a normal inhabitant of the intestine that is innately resistant to many antibacterial drugs. *Enterococcus* is the most important nosocomial Gram‐positive pathogen, except for *Staphylococcus*. NF sufu is produced using the diverse microorganisms from environment. There may be some harmful microorganisms, which could lead to food safety risks. In comparison, in MF and SF sufu, few harmful bacteria are present during fermentation (Schön et al., 2016).

The fermentation of sufu is mainly based on the synergistic action of microbial flora. Various enzymes secreted by microbial strains play important roles in complex chemical reactions. They promote the decomposition of macromolecules into small molecules and enrich the fermented product with protein, amino acids, fats, fatty acids, soy isoflavones, and other nutrients (Sun et al., 2018). Protein is an important component in all cells and tissues of the human body; it is an indispensable nutrient that should be consumed every day. Fat is a good energy storage material in cells and mainly provides heat energy. However, excessive fat intake can result in obesity and poor health. Currently, more people are pursuing the consumption of low‐fat products. EAAs and essential fatty acids are essential nutrients for growth and development. They not only promote growth and development but also reduce blood fat levels. Soybean isoflavones are typical antioxidants, and they are present in high levels in sufu, which can reduce the blood cholesterol levels and the risk of coronary heart disease.

Studies have shown that the content of nutrients differs in three types of sufu. In the current study, the fat content in NF sufu was higher than that in MF sufu and SF sufu. No difference in protein content was observed among the three types of sufu, and the ratio of fatty acids to amino acids in SF sufu was much higher than that in MF sufu. This difference was mainly due to the uniform distribution of microbial species in MF sufu; for example, *Bacillus* can produce a variety of digestive enzymes to improve the digestion and absorption of nutrients. The activities of protease, amylase, and lipase in *Bacillus* are high. During fermentation, microorganisms secrete proteases, lipases, and other enzymes. These enzymes promote the decomposition of macromolecular substances into small molecules, so the nutrients in sufu were enriched. The nutrients in NF sufu are similar to those in MF sufu. NF sufu is rich in methionine (Met), which can be converted into cysteine under the action of many types of microbial flora (Palaric et al., 2018; Speranza et al., 2017). Cysteine can damage endothelial cells in the arterial wall, leading to the deposition of cholesterol and triglycerides in the arterial wall and the development of atherosclerosis; thus, it is not safe. The nutritional quality of SF sufu was lower due to inoculation with a single strain, which inhibited the growth and reproduction of other microorganisms, and the enzyme system was relatively simple.

**FIGURE 6:**
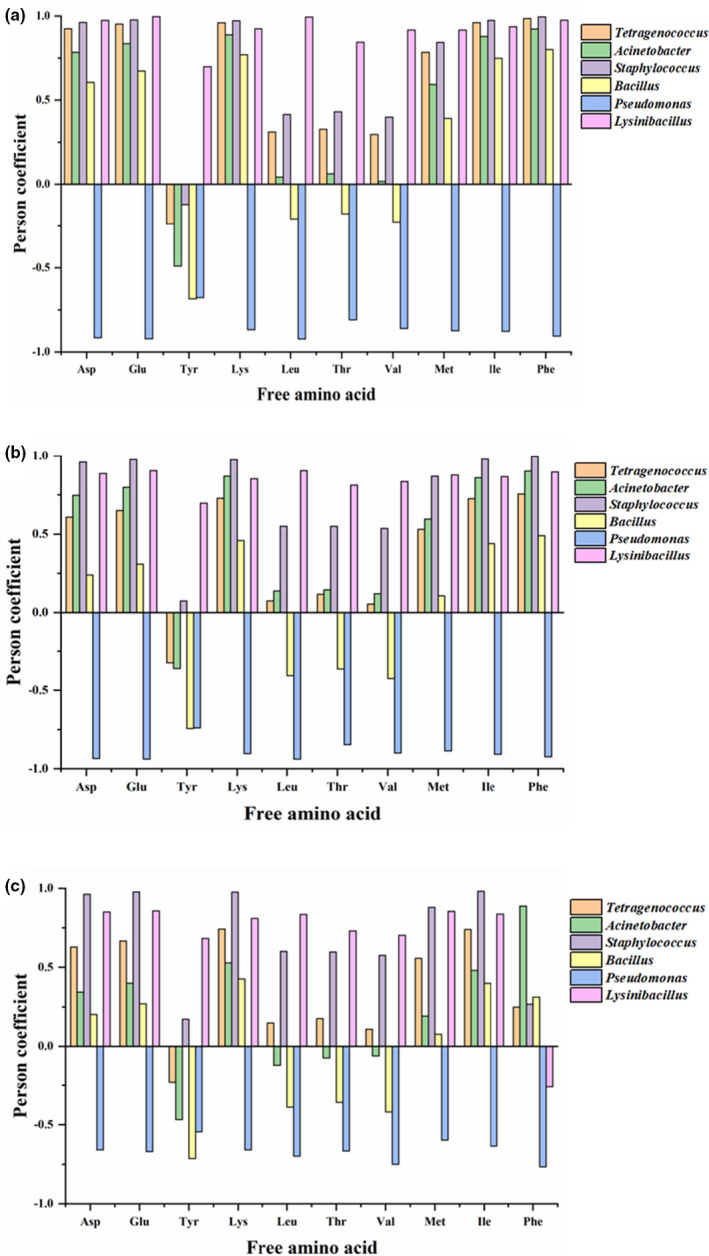
Correlation analysis between free amino acid and bacteria in sufu. (a) Correlations between six dominant bacteria and FAAs in NF sufu, (b) Correlations between six dominant bacteria and FAAs in MF sufu, (c) Correlations between six dominant bacteria and FAAs in SF sufu. The coefficients represents the significant degree of correlation between them. Free amino acid contain Asp, Glu, Tyr, Lys, Leu, Thr, Val, Met, Ile, Phe; bacteria contain *Tetragenococcus*, *Acinetobacter*, *Staphylococcus*, *Bacillus*, *Pseudomonas*, *Lysinibacillus*

To investigate the effects of bacteria on nutritional quality, we calculated Pearson's correlation coefficients of correlations between six dominant bacteria and FAAs in the three types of sufu (Figure 6). *Tetragenococcus* and *Acinetobacter* exhibited significantly positive correlations with aspartic acid (Asp), Glu, Lys, Met, isoleucine (Ile), and phenylalanine (Phe) in NF and MF sufu but considerably weaker correlations in SF sufu. *Staphylococcus* was positively correlated with Asp, Glu, Lys, Met, Ile, and Phe in NF and MF sufu. In addition, the correlations between *Staphylococcus* and Leu, threonine, and Val were enhanced in MF and SF sufu. *Bacillus* was negatively correlated with Tyr in the three types of sufu and only exhibited significantly positive correlations with Asp, Glu, Ile, and Phe in NF sufu. *Pseudomonas* exhibited a significantly negative correlation with Asp, Glu, and the other amino acids in the three types of sufu, and the correlation coefficient of *Pseudomonas* in SF sufu was the lowest. *Lysinibacillus* was positively correlated with the ten types of FAAs, except for Phe, in SF sufu. Microorganisms are key contributors to the formation of FAAs. The correlations of *Tetragenococcus* and *Acinetobacter* with Glu and Asp indicated that these microorganisms might contribute to the development of the umami flavor in sufu. Nevertheless, *Bacillus* and *Pseudomonas* may inhibit the production of various amino acids.

## 5 CONCLUSION

In this study, dynamic changes in nutrients and bacterial communities were analyzed, and the differences in nutritional quality and microbial diversity in sufu were explored. The results provide a comprehensive understanding of the biochemical process of sufu. In MF sufu, the contents of EAA and essential fatty acids are high. The distribution of bacteria and nutrients in sufu is uniform, which is beneficial to the enrichment of sufu. Using different fungi in the production of sufu is a favorable approach to improve the flavor of sufu. In addition, comprehensive studies on the correlation among microbial survival, metabolism, and flavor substances in sufu fermentation should be conducted.

## CONFLICT OF INTEREST

The authors declare no conflict of interest.

## AUTHOR CONTRIBUTIONS

**xingjiang li:** Conceptualization (equal); Funding acquisition (lead); Project administration (lead); Resources (equal); Supervision (lead); Writing‐review & editing (equal). **ying He:** Data curation (equal); Funding acquisition (equal); Investigation (equal); Writing‐original draft (equal). **wei Yang:** Software (equal). **Dongdong Mu:** Project administration (supporting). **min Zhang:** Visualization (supporting). **Yilong Dai:** Funding acquisition (equal). **Zhi Zheng:** Methodology (equal). **Shaotong Jiang:** Methodology (equal). **Xuefeng Wu:** Project administration (supporting); Visualization (lead); Writing‐review & editing (equal).

## STATEMENT OF DECLARATION FOR HUMAN SUBJECTS

This study has no involvement of human or animal subjects.

## INFORMED CONSENT

Written informed consent was obtained from all study participants.

## Supporting information

Supplementary Material

## Data Availability

The data that support the findings of this study are available from the corresponding author upon reasonable request.
