# Supplementary material for: Comparative analysis of the microbial community and nutritional quality of sufu
Source: Food Sci Nutr. 2021 Jun 23;9(8):4117–26. doi: 10.1002/fsn3.2372 (PMC8358361; doi:10.1002/fsn3.2372)
Supplement: Supplementary file 1 — Supplementary Material [file FSN3-9-4117-s001.docx]

**Supporting information**

**Figures**

**Figure A.1** Species composition in sufu at the Species level

NF: Natural fermentation, MF: Mixed fermentation, SF: Single strain fermentation. The ordinate is the sample name, the abscissa is the proportion of the species in sufu; the columns of different colors represent different species, and the length of the column represents the proportion of the species.

**Figure A.2** Texture analysis of sufu.

NF: Natural fermentation, MF: Mixed fermentation, SF: Single strain fermentation.

A: The curve of hardness during fermentation, B: The curve of adhesiveness during fermentation, C: The curve of springiness during fermentation.

**Figure A.3** Electron microscopy scan structure of sufu.

A: Natural fermentation (NF), B: Mixed fermentation (MF), C: Single strain fermentation (SF).

**Figure B.1** The white bars cultured by different fermentation methods. A: Natural fermentation (NF), B: Mixed fermentation (MF), C: Single strain fermentation (SF).

**Figure B.2** Preparation of Sufu.

A: White Bars, B: White Bars with salt, C: Aging sufu.

**Table**

**Table A.1** Primer sequence of bacteria.

**Figure A.1**

**
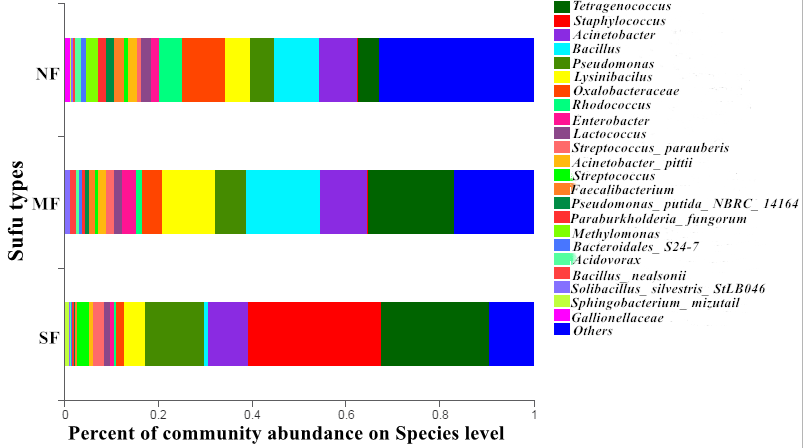
Figure A.2**


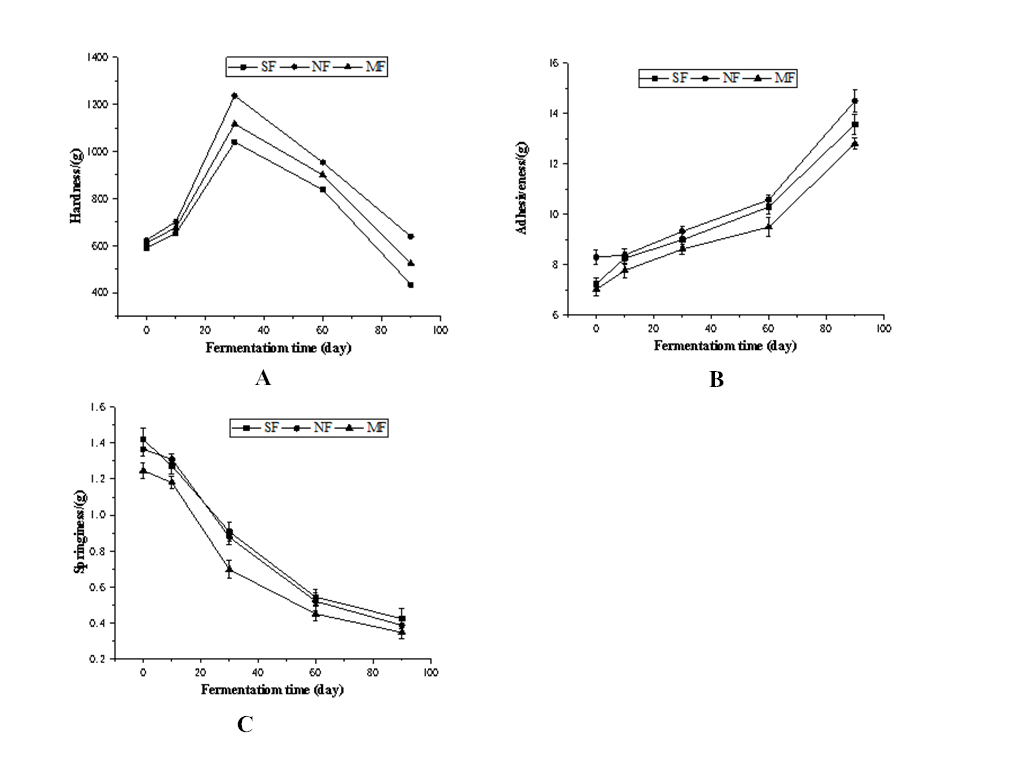


**Figure A.3**

**
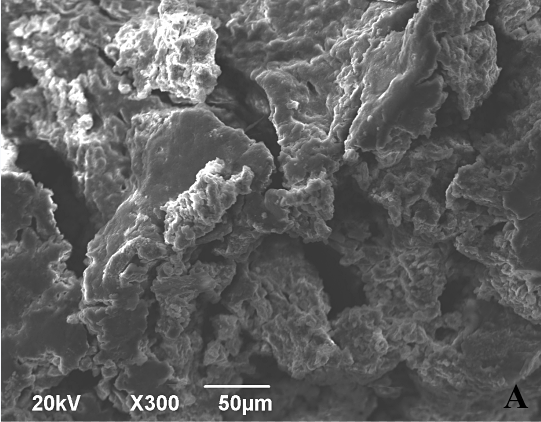
**

**
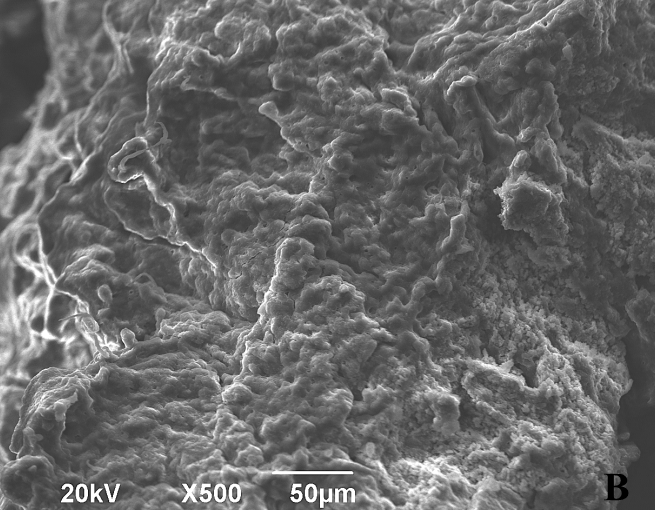
**

**
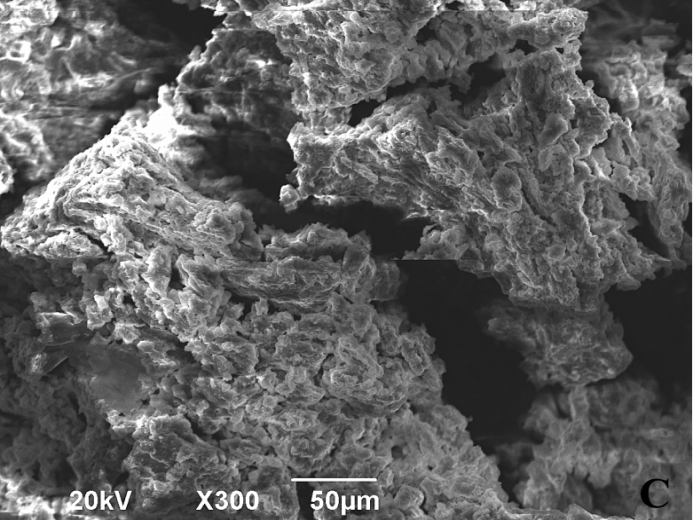
**

**Figure B.1**

**
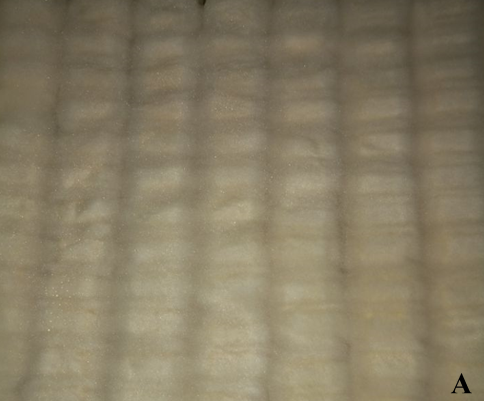
**

**
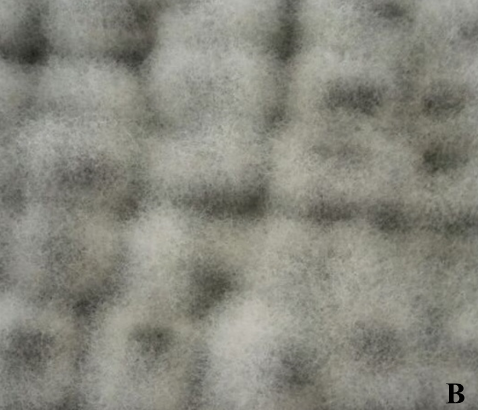
**


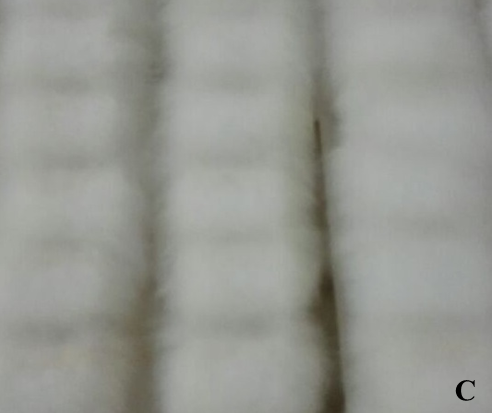


**Figure B.2**

**
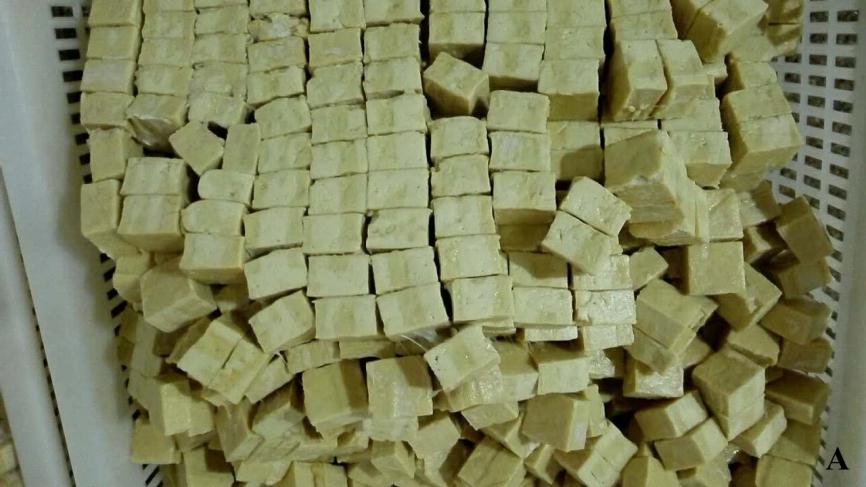

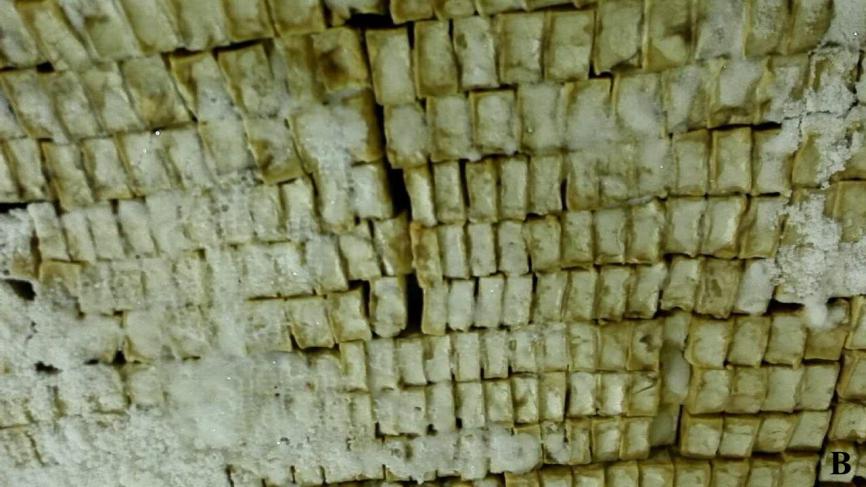

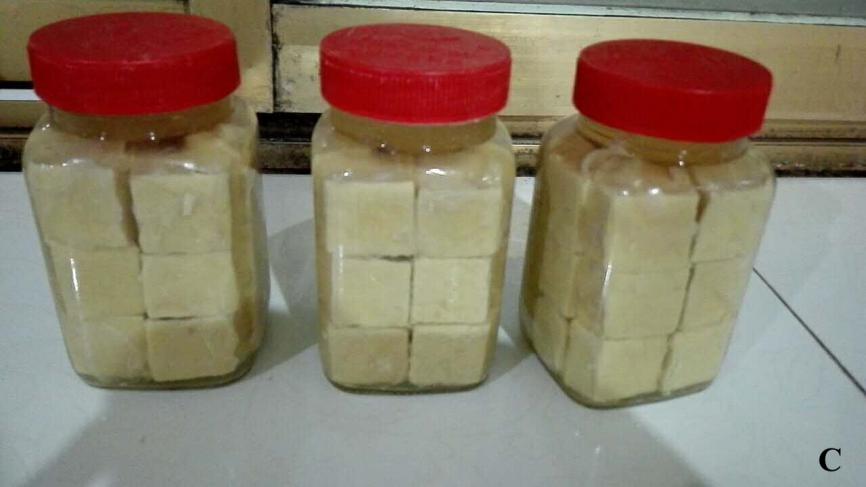
**

**Table A.1** Primer sequence

| Primer name | Primer sequence |
| --- | --- |
| 338F | 5’-ACTCCTACGGGAGGCAGCAG-3’ |
| 860R | 5’-GGACTACHVGGGTWTCTAAT-3’ |
